# Error and optimism bias regularization

**DOI:** 10.1186/s40537-023-00685-9

**Published:** 2023-01-28

**Authors:** Nassim Sohaee

**Affiliations:** Department of Information Technology and Decision Science, University of North Texas, 1155 Union Circle, Denton, TX 76203 USA

**Keywords:** Regression, Regularization, Cost function, Optimism bias, Over-estimation, Under-estimation, Convex cost function

## Abstract

In Machine Learning, prediction quality is usually measured using different techniques and evaluation methods. In the regression models, the goal is to minimize the distance between the actual and predicted value. This error evaluation technique lacks a detailed evaluation of the type of errors that occur on specific data. This paper will introduce a simple regularization term to manage the number of over-predicted/under-predicted instances in a regression model.

## Introduction

The main focus of machine and statistical learning models is on developing reliable predictive models based on available data. In modern machine learning, regularization is a common practice to control the ability of a model to generalize to new settings by trading off the model’s complexity. Model regularization is a simple yet efficient way to compute model parameters in the presence of constraints to control model complexity. Specifically, regression regularization is an established method of increasing prediction accuracy in many regression models. Model complexity in regression learning models is displayed in high prediction variability. The regularization terms aim to control the prediction variability with a slight increase in bias.

Over or under-estimation is a real challenge in many data science applications, from business to life science, to derive a reliable prediction generated by machine learning models. For example, as a social and business science, price science uses economics, statistics, econometrics, and mathematical models to study the problem of setting prices. In finding the optimal price to maximize profit/revenue with statistical and machine learning models, when the size of available products increases, the predicted gross profit tends to overestimate [1]. In another instance, India’s Central Statistics Organization (CSO) changes its method of computation of national income due to the overestimation in the growth rates [2]. The challenges with over/underestimation in prediction are not limited to business or industry. In biology, scientists observed that machine learning models tend to overestimate the protein–protein association rates [3]. In medicine, the statistical and machine learning models missed the pain level of about 50 percent of women during labor [4]. Labor pain prediction is vital in obstetrics and helps caregivers properly manage the pain. The over/under-estimation may cause a significant interruption in future applications. So, besides minimizing the difference in the prediction loss, we should bring attention to another form of model complexity that results in over/under-estimation.

In many machine learning applications, underpredicting or overpredicting is not realized until the estimated period is over. An overprediction or overestimation predicts that the estimated value is above the realized value. On the other hand, underprediction or underestimation indicates that the predicted value is below the actual value. Even if you observe an over or underprediction in the results, the learning model cannot address it systematically by model refinement and hyperparameter setting.

In most regression models, the estimation and inferences are closely tied with the prediction variance. Generally, when the model generates a high variance, we can assume a large information loss is happening [5]. To avoid high variance in regression prediction, we generally modify the cost function of a regression model to penalize large model coefficients for lowering the variance at the cost of increased biased. The well-known approaches are LASSO and Ridge. LASSO [6] or variations of ${\mathcal{l}}_{1}$ canalization [7], or even a costume ${\mathcal{l}}_{1}$ penalty function set to bound the magnitude of the control coefficients [5]. Ridge [8] or ${\mathcal{l}}_{2}$ penalty is usually added to the cost function to address the collinearity problem in linear regression models. Many machine learning models with Least Squared Error as a cost function may provide a basis for the overall prediction. Still, collinearity will cause an improper weight assignment to available variables. Theobald [9] extended this result to a more general loss function.

The quality of a regression model is measured by the discrepancy or loss between the actual output and the estimated output. The goal of learning is to find a model with minimal prediction error. It is unknown that machine learning models with convex loss function have an optimum complexity that generates the smallest prediction error [10]. Therefore, most of the models have some provisions for complexity control. Usually, we use regularization as an effective method to control the model complexity [11]. The problem set we have in this paper aims to reduce the number of instances with over or under-estimated predictions. The novelty of this paper lies in two parts. First, we introduce a regularization term that can be added to the cost function of any machine learning model with a convex cost function. The optimism bias regularization term provides a satisfactory theoretical and conceptual framework for learning with the finite sample. We know that sometimes theoretical concepts may not translate into a practical application. In the second part of this paper, we demonstrate the valuable capability of using optimism bias to control the complexity of regression models. For this purpose, we test regularization terms on various regression models both on synthetic and real-world data.

This paper is organized as follows. “Problem definition” section describes classical model selection criteria in statistics and machine learning. In this section, I will introduce the optimism bias regularization term. “Numerical results” section describes the empirical comparisons between regression models and regularized regression models. “Experiments” section implies the optimism bias regularization on the Covid-19 data set to discuss the real-world application of the proposed regularization term. The application of optimism bias regularization is not limited to only business focus data sets. The Covid-19 case study shows how this regularization term affects the performance of predictive models in a broad range of applications form business to science.

## Problem definition

The predictive learning algorithms have a similar goal of generalizing the learning from the random sample to the entire population. We can achieve this goal by minimizing the prediction risk. The quality of a machine learning model is measured by the discrepancy or loss function $L(y,h(X))$ between the actual output and its estimate produced by the machine learning model [12]. Hence, the goal of learning is to find the best function *h* from a given set of functions (or models), and this can asymptotically be possible if the goal is an accurate function approximation [11, 13]. Regression model generalization can be decomposed into three components: bias, variance, and noise. The bias-variance tuple demonstrates the complexity of the learning model. As model complexity increases significantly, the model variance may also be elevated while the bias gets smaller. This bias-variance behavior is a sign of over-fitting, and the trade-off is known as a generalization error. Regularization is a key component in machine learning to overcome the fundamental issue of over-fitting problems [14].

Poggio and Girosi [15] established an application of regularization theory for machine learning models with finite train data sets. The traditional regularization approach was to find a continuous function $\Psi$ to perform a one-to-one map from a normed space $X$ onto another normed space $y$. Data scientists use different non-negative penalty functions on various machine learning models. The primary goal of regularization is to find a function $h(X)$ for independent variable $y(X)$ that minimizes the functional:

$$L\left(y,h\left(X\right)\right)+ \beta \Psi \left(h\left(X\right)\right),$$

where $\Psi$ is the non-negative regularization function which penalizes the function $h$ with the regularization parameter $\beta$.

### Optimism bias control

We will present an optimism bias control regularization for regression machine learning models with convex loss function, including linear regression, SVM, and deep learning models. There are many efficient approaches to minimize the convex and continuous loss functions. In this setting, we can formulate the supervised regression model as follows:

$$h\left(x\right)= W.\Phi \left(X\right)+ b.$$

where Φ is a mapping function that can be considered kernel transformation. The solution to the Empirical Risk Minimization (ERM) optimization problem can be described as

$$h:\mathrm{arg}\underset{W,b}{\mathrm{min}}\widehat{L}(y, h\left(X\right))$$

where $\widehat{L}$ is the generalization error of the model that can be represented as

$$\widehat{L}\left(h\right)=\frac{1}{n}\sum_{i}\mathcal{l}({y}_{i}, h\left({X}_{i}\right)).$$

Unfortunately, ERM minimization tends to result in over-fitting in many applications. To avoid over-fitting, we can apply Tikhonov or Ivanov regularization techniques [16].

$$h:\mathrm{arg}\underset{W,b}{\mathrm{min}}\widehat{L}\left(y,h\left(X\right)\right)+\lambda .P(W)$$

The regularization term $P(W)$ is defined to stabilize the estimates by adding a penalty to the empirical risk. The penalty multiplier $\lambda$, ranging from $0$ to $+\infty$, controls the degree of stabilization. Two widely used $P$ functions are famously known as ${\mathcal{l}}_{2}$ and ${\mathcal{l}}_{1}$ regularization terms. Two functions increasingly penalize large absolute values for the feature coefficients in different manners [17]. However, neither of these models can help control a specific type of regression prediction error. For this purpose, we introduce a new regularization term to target a specific type of prediction in a regression model. The control optimism penalty can be formulated as:

$$h: \text{arg }{\text{min}}_{W, b} \widehat{L}\left(y, h\left(X\right)\right)+\frac{\beta }{2}\Psi \left(h\left(X\right)\right),$$

where

1 $$\Psi (h(X)) = \sum_{i}(sgn(h(Xi) - yi) + 1).\mathcal{l}p(h(Xi) - yi)$$

or

2 $$\Psi (h(X)) = \sum_{i}(-sgn(h(Xi) - yi) + 1).\mathcal{l}p(h(Xi) - yi)$$

Respectively, to regularize overprediction or underprediction in regression models. The resulting regularized cost function is a strictly convex function that grants the existence of an optimal solution.

#### **Lemma 1**

$\alpha f$* is convex if f is convex and* $\alpha \ge 0$.

#### **Lemma 2**

*The sum of two convex functions is strictly convex if at least one is strictly convex.*

#### **Theorem 1**

$\Psi \left(h\left(X\right)\right)= \frac{\beta }{2} \sum_{i}\left(sgn\left(h\left({X}_{i}\right)-{y}_{i}\right)+1\right).{\mathcal{l}}_{p}\left(h\left({X}_{i}\right)-{y}_{i}\right)$ *is a convex function for* $\beta \ge 0$ *and *$p\ge 1$*.*

#### *Proof*

$-1 \le sgn(h(Xi)-yi) \le 1\, for \, \forall \, Xi \in Xtrain$Therefore, $0\le \frac{\beta }{2}sgn\left(h\left({X}_{i}\right)-{y}_{i}\right)\le \beta$, which is a non-negative number. Also, any *ℓ*_*p*_ is a strictly convex function for $p\ge 1$. Hence, $\Psi (h\left(X\right))$ is a convex function. □

Similarly, we can prove that $\Psi (h(X)) = \sum_{i}(-sgn(h(Xi)-yi)+1).{\mathcal{l}}_{p}(h(Xi)- yi)$ is also a convex function.

#### **Theorem 2**

*Assume* $\widehat{L}\left(y, h\left(X\right)\right)$ *is a strictly convex function, then* $\widehat{L}\left(y,h\left(X\right)\right)+\frac{\beta }{2}\Psi (h\left(X\right))$ *is a strictly convex function for* $p>1$ *.*

The proof of the above theorem is trivial considering the Theorem 1 and Lemma 2. In this paper $p = 2$ in the ${\mathcal{l}}_{p}$ norms. Hence, an optimism-biased regularized cost function is still strictly convex and can be regularized further for model complexity control using a ${\mathcal{l}}_{p}$ for $p\ge 1$. The existence theorem guarantees a global minimum for the regularized cost function.

## Numerical results

This section presents numerical results from simulation studies and a real-world data analysis to study the performance of Optimism Bias regularization in the context of different models. The simulation scenarios include univariate and multi-variant linear regression models, support vector regression models, and deep learning models. It’s worth stating that the prediction errors in Machine Learning models are categorized as reducible and irreducible errors. Irreducible error is the natural result of variability in the system, which suggests that the model may not change this type of error. On the other hand, the reducible error can be represented with two statistical functions, bias, and variance, to display the complexity and flexibility of the model for the available training data set. In our study, we will use bias and variance to study the complexity and flexibility of each model with optimism bias regularization term.

### Linear regression models

**Fig. 1:**
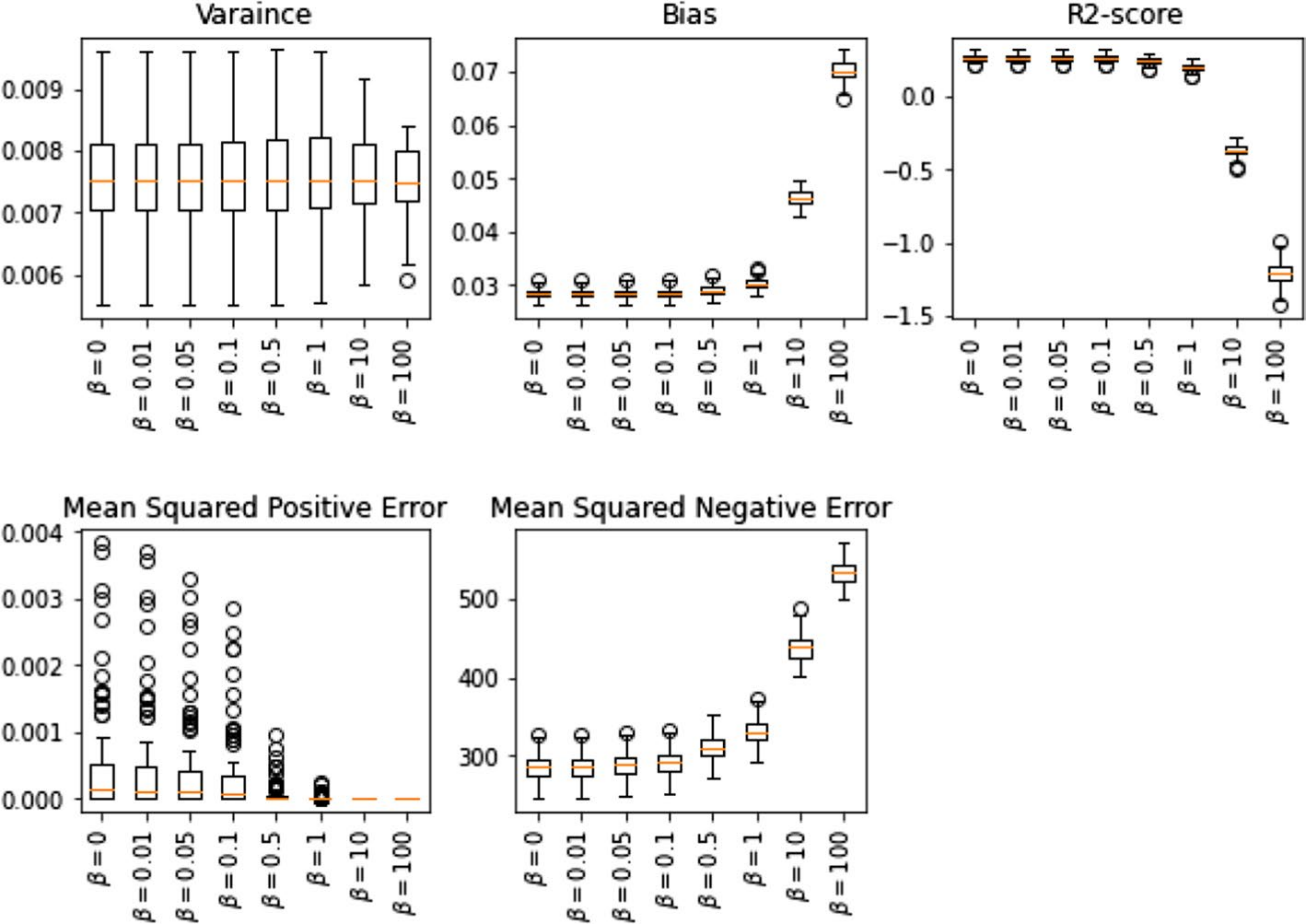
The effect of hyperparameter β on variance, bias, and score of univariate linear regression

For the first simulation, we consider the univariate linear regression model. We are interested in seeing the effect of the regularization hyper-parameter $\beta$ on variance, bias, and regression score and in controlling the optimism bias. For this purpose, we have generated one hundred random univariate linear regression data sets, each with one thousand instances. The box plots Fig. 1 display, on average, the model variance will have minor changes as the value of the hyper-parameter $\beta$ increases. On the other hand, bias will rapidly increase for larger hyper-parameter $\beta$ values, which suggests the regularized method tends to underfit. The sharp drop in the average r2-score suggests that increasing $\beta$ may result in over-fitting. Meanwhile, by adding this regularization term, we can control the means squared positive and negative errors.

**Fig. 2:**
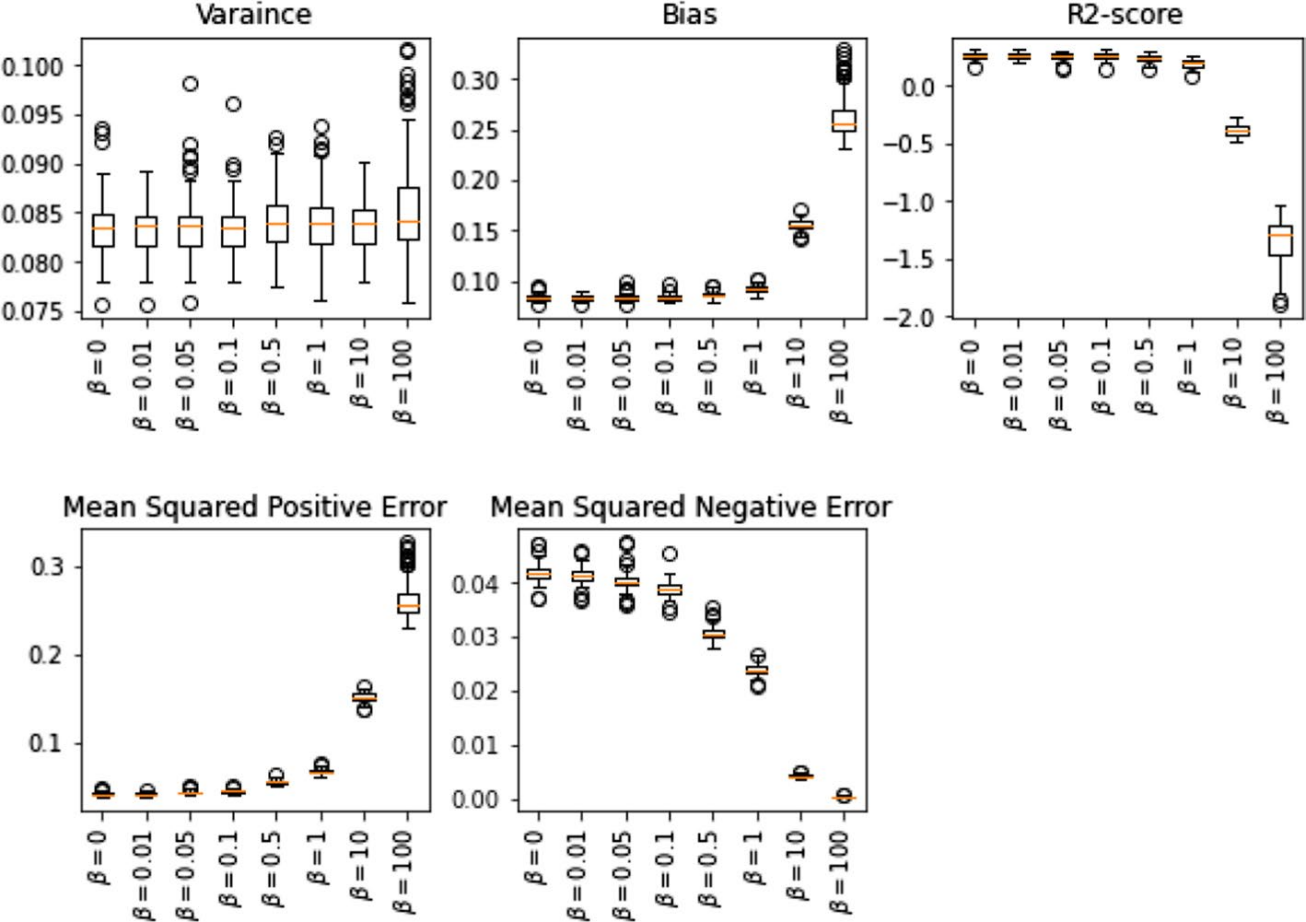
The effect of hyperparameter β on variance, bias, and score of multivariate linear regression

We tested the new regularization term on a multivariate linear regression model in the second simulation. We have generated random data sets for this simulation with one thousand instances and four features. The results are summarized in Fig. 2. The observation suggests that when the value of hyperparameter $\beta$ grows, in average variance does not significantly change while bias increases and regression score declines. Also, as it is expected, the mean squared positive error increases while the mean squared negative error decreases. For this implementation, we considered Eq. 1. If we consider Eq. 2, then we are expected observe the opposite behavior on mean squared positive and negative errors.

### Support Vector Machine Models

The Support Vector Machine (SVM) model was introduced by Boser et al. [18] and immediately became one of the most successful algorithms for machine learning problems. Vapnik initially studied the approach, and Lerner [19] and Vapnik and Chervonekis [20] as an alternative statistical learning model even though the initial algorithm focused on classification problems, but soon after, many scientists observed the excellent performance of regression and time series applications. The support vector regression problem can be expressed as

$$\mathrm{min}\frac{1}{2}{\omega }^{2}+C\sum_{i=1}^{n}({\xi }_{i}+{\xi }_{i}^{*})$$

$$\text{subject to }\left\{\begin{array}{c}{y}_{i}-h\left({X}_{i}\right)\le \epsilon + {\xi }_{i}^{+}\\ h\left({X}_{i}\right)-{y}_{i}\le \epsilon + {\xi }_{i}^{-}\\ {\xi }_{i}^{+}, {\xi }_{i}^{-}\ge 0\end{array}\right.$$

where *ξ*_*i*_^+^ and *ξ*_*i*_^−^ are slack variables representing upper and lower constraints on the system’s outputs, and $C$ is the regularization coefficient controlling errors’ influence. This loss function is considered an *ϵ*-insensitive loss function, which means errors less than *ϵ* are ignored in the process. The function *h* could be the linear function or any other non-linear function to address the curse of dimensionality, known as the kernel trick.

**Fig. 3:**
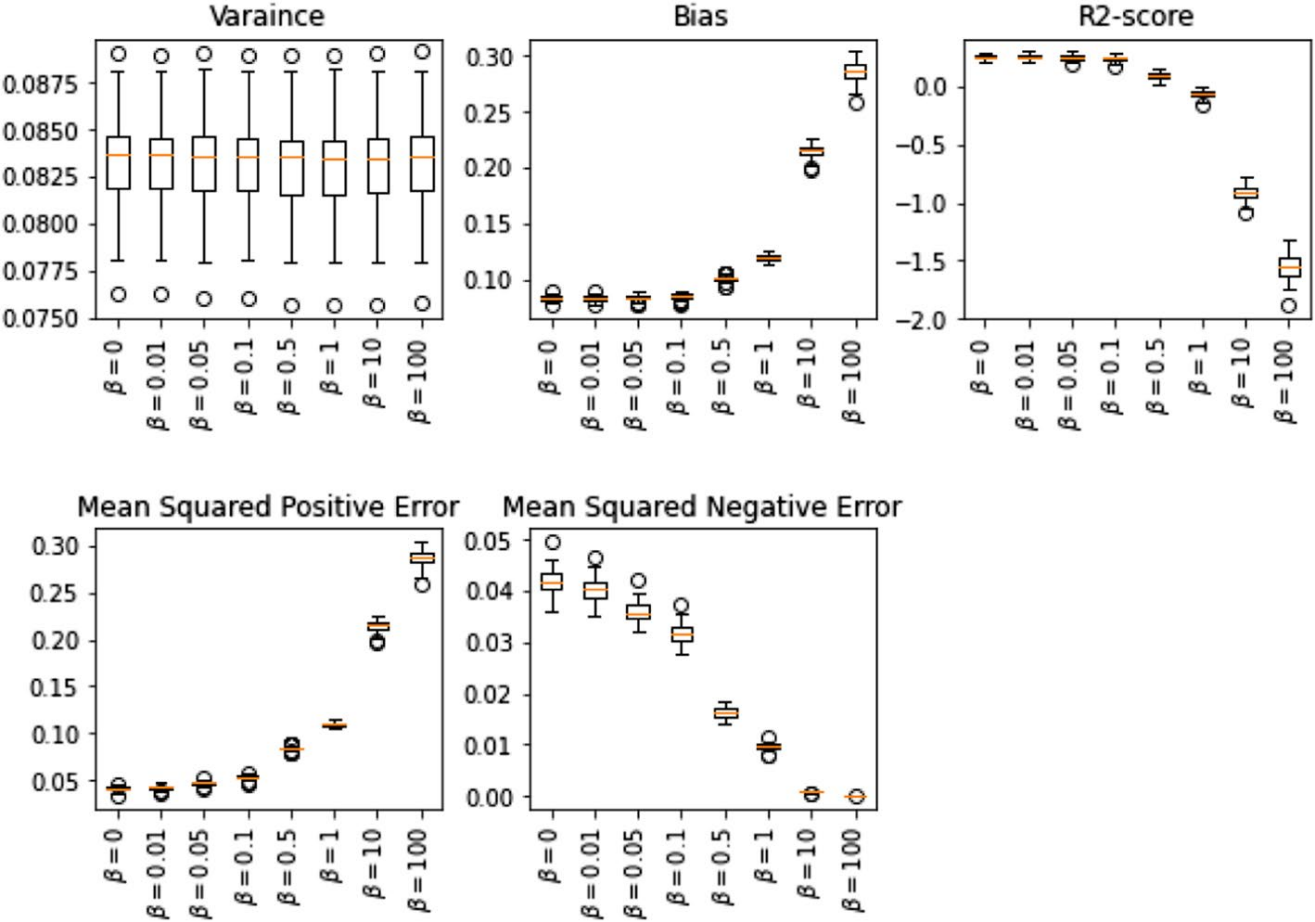
The effect of hyperparameter β on variance, bias, and score of multivariate Support Vector Machine Regression

Similarly, we can modify the SVM problem to consider and control a specific error by adding a new regularization term $\frac{\beta }{2}\Psi (h\left(X\right))$ to the optimization problem, where $\Psi$ could be Eqs. 1 or 2 depends on the objective of the problem. We have used a similar multivariate data set to study the effect of added regularization terms. Figure 3 illustrates similar results to the linear regression models, proving that the intended changes limit the specific over/under prediction in a random data set.

## Experiments

The emergence of coronavirus disease 2019 (COVID-19) has quickly become the major healthcare threat in 2020. Our understanding of this deadly virus and possible treatment methods is changing daily. In the United States, the disease is expected to infect 20 to 60 percent of the population [21]. The recent experiences in New York and other parts of the world in handling COVID-19 spikes strongly suggest that a critical factor in keeping the number of deaths low is to ensure the adequate capacity of inpatient and ICU beds, especially during the surge.

Any outbreak of an epidemic requires an ample amount of historic data to learn and make future forecasts. The unique nature of each outbreak is a factor against the accurate prediction. Even though we might have much available historic data on similar outbreaks, each epidemic outbreak is still unique, making the available data irrelevant.

One of the significant challenges in the early COVID-19 pandemic was the lack of preparation for surges. Optimism bias prediction may have many applications in regular and cross-sectional data sets. Similarly, there are some significant use cases in the times series.

### Covid-19 case study

In this experiment, we have used the Covid-19 patient data collected in the state of Washington since the early days of the outbreak. Our study shows that simple predictive methods like moving averages and more complex approaches like Long Short-Term Memory (LSTM) [22] cannot predict a surge quickly. As a result, they cannot be used as a basis for medical care preparation. LSTM is a widely used recurrent neural network for processing sequential data such as time-series data. In a time series prediction, the goal is to forecast the upcoming trends/patterns given historical data sets with temporal features. Recurrent Neural Networks (RNN) are great tools in sequence data analysis and prediction [23]. The major shortcoming of RNN is dealing with long distance dependencies. LSTM, a special kind of RNN network, establishes a long delay between input, feedback, and gradient explosion prevention to overcome this issue.

This paper will use the LSTM model with optimism bias regularization to predict the number of Covid-19 cases in the recent world pandemic. Because Covid-19 has caused a significant interruption in human life worldwide, strategic planning in the public health system to avoid deaths and manage patients is a crustal task. There is plenty of research conducted on this topic since the emergence of this pandemic to forecast the number of Covid-19 cases using the LSTM or a variation of this model [21, 24, 25]. Shahid et al. [25] compared LSTM with supervised learning models like auto-regression integrated moving average (ARIMA), support vector regression (SVR) for time series prediction of confirmed cases, deaths, and recoveries in ten major countries affected to Covid-19. The comprehensive comparison shows that LSTM outperforms the other two models. Therefore, for the last case study of this paper, I apply the LSTM model with the new optimism bias regularization to forecast the number of cases affected by Covid-19.

Data used for this case study is collected on the last day of August 2021 from the [CDC data tracker]. We have trained the LSTM model and LSTM with optimism bias regularization terms. We tested a different range of beta hyper-parameter in this case study. This paper aims to show how optimism bias regularization affects prediction quality and can achieve the previously impossible goal through model refinement.

**Fig. 4:**
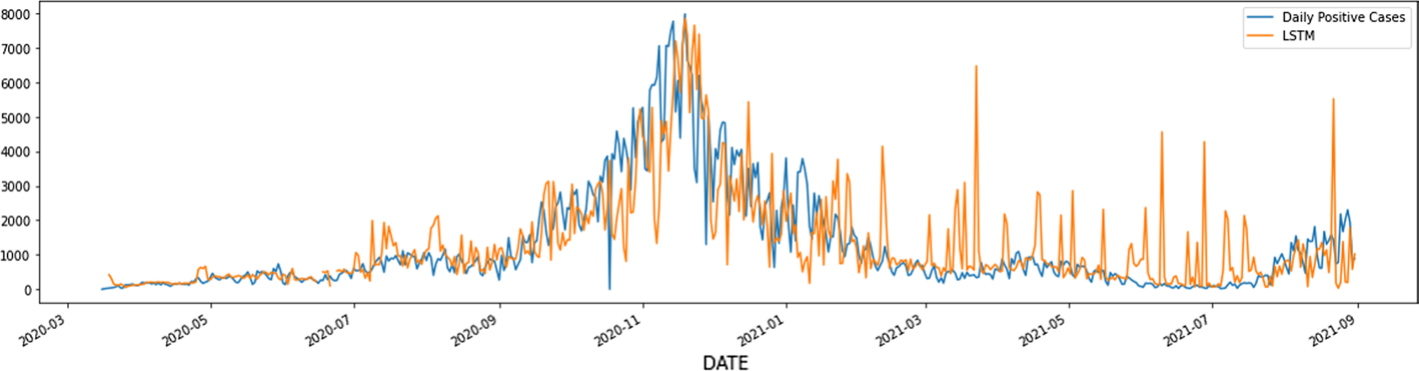
Forecasting the number of daily positive cases in the state of Washington

**Fig. 5:**
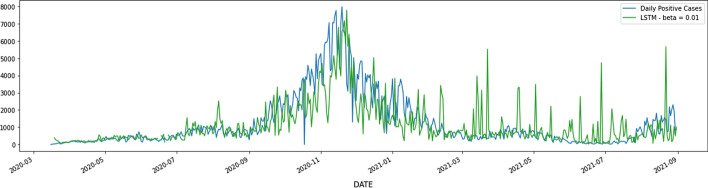
Forecasting the number of daily positive cases in the state of Washington using the LSTM model with optimism bias regularization term

Figure 4 shows a daily number of positive cases of Covid-19 in the state of Washington and LSTM predictions. As this figure illustrates, during the surges LSTM model is under-predicting the daily number of positive cases. Therefore cannot be a reliable source for the state and the hospital to prepare themselves for the surge. Figure 5 displays the prediction made by an LSTM model enhanced with an optimism bias regularization and regularization parameter 0.01. According to Table 1, when hyperparameter *β* = 0*.*01, values of Mean Squared Error and Mean Positive Squared Error improves. This fact suggests that the LSTM model with optimism bias regularization term *β* = 0*.*01 better captures the magnitude of the catastrophe during the surge. LSTM model without optimism-biased regularization under-predicts the daily number of positive cases in 295 days out of 534 days considered in this study. LSTM with optimism-biased regularization and regularization coefficient *β* = 0*.*01 cuts the number of days with under-estimated positive cases by half to 173 days. This significant drop directly affects medical facility readiness, especially during the surge time.

**Table 1:** Hyperparameter tuning in Covid-19 daily positive case study

Model	Mean squared error	Mean positive squared error
LSTM	557,289.587901	6.965658e + 05
LSTM—*β* = 0*.*01	537,408.970020	6.710319e + 05
LSTM—*β* = 0*.*1	527,946.192856	8.641470e + 05
LSTM—*β* = 0*.*8	677,850.136593	8.442096e + 05
LSTM—*β* = 1	530,538.572982	1.256760e + 06

## Data driven remaining useful life prediction

The remaining useful life (RUL) is when a machine is likely to operate before it requires repair or replacement. The application of RUL is from material science to biostatistics and econometrics. RUL estimation is one of the main challenges in conditional-based maintenance and prognostic health management. Method-based RUL prediction models build mathematical models to explore the degradation trajectory of machinery. The benefit of these types of models is their high accuracy. However, as mechanical systems’ complexity increases, such models’ design and implementation may result in extraordinarily complex and sometimes impossible-to-solve mathematical models. Also, the model development heavily depends on domain expert knowledge of system components.

Alternatively, a data-driven approach estimates RUL using sensor and operational data and traditional machine learning models. Data-driven models rely on previously observed data to predict the systems’ future state. Researchers applied various machine learning models like polynomial regression, support vector regression, and autoregressive integrated moving average models to forecast RUL. Deep neural network techniques outperform other machine learning models when the complexity of the mechanical system increases and available sample data suggest a robust nonlinear pattern [26–28].

For the second case study, we use Commercial Modular Aero-Propulsion System Simulation (CMAPPS) developed by NASA to study engine degradation [29]. This data set consists of multiple multivariate time series collected from different engines. Each engine starts with different degrees of initial wear and manufacturing variation, which is unknown to the user. Each engine usually operates at the start of each time series and develops a fault at some point. The objective of this data set is to predict the number of operational cycles after the last cycle that the engine will continue to operate.

**Fig. 6:**
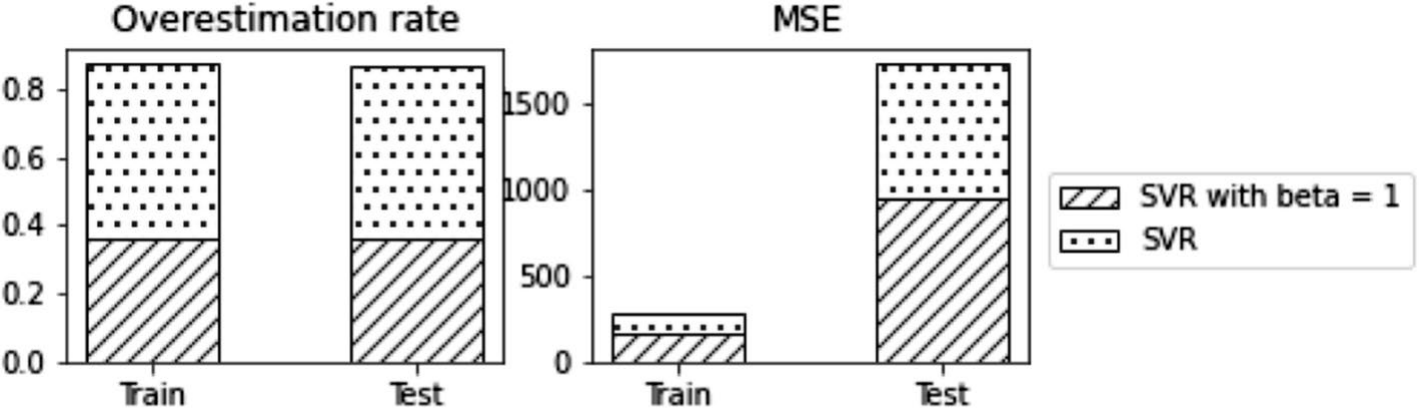
Over-estimation rate and MSE of SVR model with and without Optimization Bias Regularization trained on CMAPPS data set

We followed the data preparation technique outlined in [30] to prepare and scale the data set. The initial collected data set contains 26 columns containing information about the engine operational settings and sensor measurements of 100 engines. This data set does not provide RUL directly as a value. Instead, it has time series information of all runs to failure of all engines. From this data, we can compute the linear degradation model of each engine in this data set. RUL in this data set ranges from 128 to 362 cycles without engine failure. We consider only the sensor measurements at the beginning of each cycle for the machine learning model to predict the RUL. We use a support vector regressor model, SVR, with RBF kernel to predict the RUL of the CMAPPS data set. The computed best parameters for this model are *C* = 10 and *ϵ* = 5. However, this model overestimates the RUL of about half of the instances in both train and test data sets. Considering the cost of over-estimation, it is essential to build a model that, while minimizing the prediction cost, also avoids overestimation as much as possible. For this purpose, we have trained an SVR model with optimism-biased regularization. The overestimation in this data set is costly and may cause several operational obstacles in the system. To control the number of instances with RUL prediction higher than the actual value, we have included the optimism regularization term with regularization coefficient *β* = 1. Figure 6 compares the RUL predictions with and without optimism bias regularization. The overestimation rate in this plot is the ratio of instances with RUL predicted value higher than the actual to the total number of instances. For the regularization parameter *β* = 1 we could control the over-estimation by 40 percent from 0.5 to close to 0.3. The drawback of this significant drop in the over-estimation rate is the increase in model complexity which has been displayed in plot MSE of Fig. 6.

## Conclusion

This paper discussed a new regularization term that can be added to a convex cost function of a machine learning model. Generally, model complexity represents itself in the form of high variance in regression machine learning models. To avoid model complexity in regression prediction, We modify the cost function to penalize model coefficients. The cost of this modification usually is higher biased in prediction. Then we introduced a new function to penalize the over or underpredictions in the learning model. This new penalty function works best on machine learning models with a convex cost function. We have tested it on various synthetic and real data to validate our proposed method. In all the examples, we can inspect the rise of bias when the regularization coefficient increases. We can conclude the proposed regularization is controlling the over or underestimation complexity of the machine learning model.

## Data Availability

Data sets analyzed during current study are available in the: CDC data tracker: https://covid.cdc.gov/covid-data-tracker/#compare-trends_comptrends-cases-new-raw-lin. CMAPSS Jet Engine Simulated Data: https://data.nasa.gov/Aerospace/CMAPSS-Jet-Engine-Simulated-Data/ff5v-kuh6.
